# Supplementary material for: In slow motion: radula motion pattern and forces exerted to the substrate in the land snail Cornu aspersum (Mollusca, Gastropoda) during feeding
Source: R Soc Open Sci. 2019 Jul 3;6(7):190222. doi: 10.1098/rsos.190222 (PMC6689628; doi:10.1098/rsos.190222)
Supplement: Supplementary Figures and Table [file rsos190222supp2.docx]

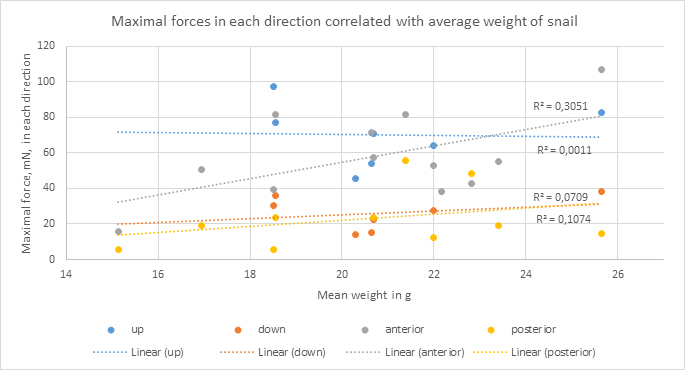


**Supplementary Fig. 1.** Maximal forces in each direction correlated with average weight of snail; no correlations were detected using linear regression between weight of snail and maximal force.


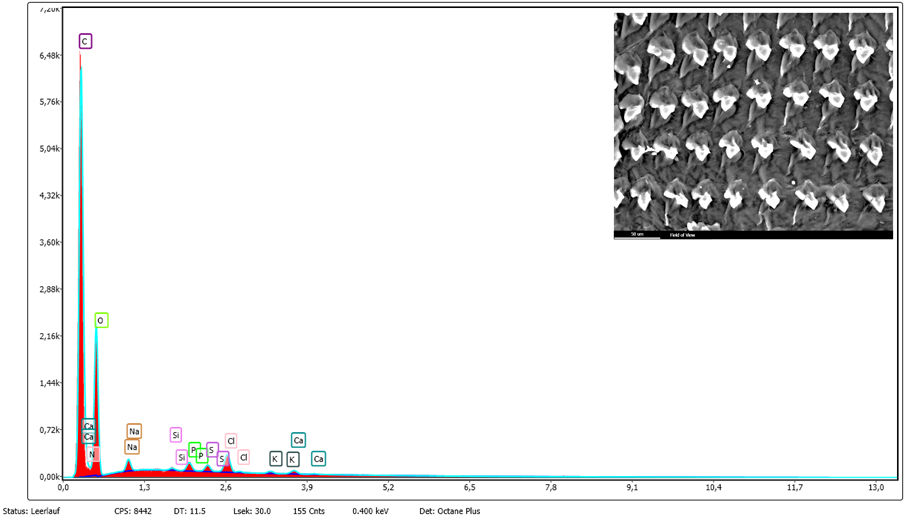


**Supplementary Fig. 2.** EDAX analysis of used marginal teeth from the depicted area for ZMH 150005-6.

**Supplementary Tab. 1.** P-values from Spearman revealed only correlation in snail ZMH 150005-14.

| p-values from Spearman | | | | |
| --- | --- | --- | --- | --- |
|  |  | Direction of force: | | |
| Snail no. | Comparison to | Up | Down | Anterior |
| ZMH 150005-1 | Down | 0,2333 |  |  |
|  | Anterior | 0,4557 | 1 |  |
| ZMH 150005-2 | Down | 0,6134 |  |  |
|  | Anterior | 0,1247 | 0,4366 |  |
|  | Posterior | 0,95 | 0,95 | 0,6833 |
| ZMH 150005-3 | Down | 0,6153 |  |  |
|  | Anterior | 0,9194 | 0,8028 |  |
| ZMH 150005-8 | Down | 0,5167 |  |  |
|  | Anterior | 0,175 | 0,7833 |  |
| ZMH 150005-9 | Down | 0,6646 |  |  |
|  | Anterior | 0,5364 | 0,3871 |  |
| ZMH 150005-14 | Down | 0,3001 |  |  |
|  | Anterior | 0,45 | 0,004818  Rho = 0,97 |  |
